# Solubility of proteins

**DOI:** 10.5599/admet.831

**Published:** 2020-06-28

**Authors:** Mauno Vihinen

**Affiliations:** Protein Structure and Bioinformatics, Department of Experimental Medical Science, BMC B13, SE-22184 Lund, Sweden

**Keywords:** aggregation, protein engineering, biologic, solubility prediction

## Abstract

Solubility is a fundamental protein property that has important connotations for therapeutics and use in diagnosis. Solubility of many proteins is low and affect heterologous overexpression of proteins, formulation of products and their stability. Two processes are related to soluble and solid phase relations. Solubility refers to the process where proteins have correctly folded structure, whereas aggregation is related to the formation of fibrils, oligomers or amorphous particles. Both processes are related to some diseases. Amyloid fibril formation is one of the characteristic features in several neurodegenerative diseases, but it is related to many other diseases, including cancers. Severe complex V deficiency and cataract are examples of diseases due to reduced protein solubility. Methods and approaches are described for prediction of protein solubility and aggregation, as well as predictions of consequences of amino acid substitutions. Finally, protein engineering solutions are discussed. Protein solubility can be increased, although such alterations are relatively rare and can lead to trade-off with some other properties. The aggregation prediction methods mainly aim to detect aggregation-prone sequence patches and then making them more soluble. The solubility predictors utilize a wide spectrum of features.

## Introduction

**Figure 1.**
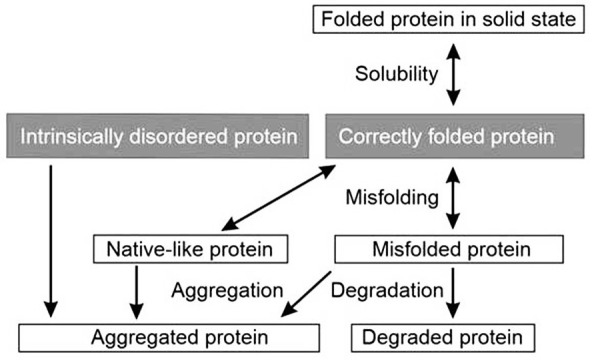
Relationships of protein solubility, aggregation and degradation. Insoluble protein is correctly folded, whereas the structure of aggregated protein is altered. Most irreversibly misfolded proteins are degraded as part of normal protein turnover. Intrinsically disordered proteins and native-like structures have their specific aggregation mechanisms.

Solubility is an important property for all drugs, including biologics. Many proteins and polypeptides are poorly soluble and those trespassing through cellular membranes, membrane proteins, are not in traditional sense soluble at all. Proteome-wide analysis of solubility in *Caenorhabditis elegans* indicated that about 75% of proteins appear in cells in abundances close to their solubility limits [1]. We can distinguish two phenomena in relation to protein behaviour in solution. Solubility and aggregation are related but have different meanings. Solubility is defined here and in many other publications as the concentration in which intact protein is in equilibrium with solid phase [2-4]. In the case of aggregation, protein molecules bind together, often due to irreversibly altered conformation, and form insoluble high molecular weight forms (see Figure 1) [5].

Unlike aggregated forms, precipitated solubilizable protein in solid phase can be made soluble by dilution. Aggregated protein is in solid phase and typically undergoes irreversible structural changes, thus aggregated protein cannot be returned back to soluble form and original structure. Proteins in these amyloid fibrils have extensive β-strand secondary structures. When intrinsically disordered proteins aggregate they form amorphous deposits, whereas native-like structures aggregate to native-like deposits (Figure 1) [5]. Both solubility and aggregation have biological and biotechnological consequences. Reduced solubility as well as increased aggregation are related to several diseases. Plaques formed by aggregated proteins are common in neurodegenerative diseases. Altered conformations of prion proteins can “infect” other proteins and cause their aggregation. β-sheet formation is often related to aggregation and prion formation. These secondary structural elements can stack intra- and intermolecularly to form large insoluble aggregates. Even protein crystallization is related to solubility. Ordered protein crystals are needed for X-ray crystallography to reveal protein structures. These crystals are grown slowly and contain typically large amounts of solvent. The goal of the crystallization is to keep the proteins in their native conformation, however certain packing effects affecting local conformation are common.

Many factors affect solubility and aggregation, including intrinsic properties of the protein, solvent and additives as well as physical conditions. List of relevant protein factors is long and could be started with amino acid sequence and composition, three-dimensional structure and exposure of residues, intramolecular interactions within the protein (salt bridges, hydrogen bonds, electrostatic and hydrophobic interactions, multimeric status etc.), and protonation status. Solvent properties (polarity, bond and interaction forming ability, density etc.) and constituents (such as excipients, salts or organic solvents) and their concentrations have significant contributions to solubility. Further, factors like pH and temperature affect both the protein and solvent.

Proteins are large and relatively fragile molecules, thus not necessarily ideal as drugs. Lots of research has been devoted to find smaller structures having (some) functions of the biological proteins. Miniproteins or protein scaffolds have been tested as biologics [6]. Some functions can be retained even in short peptides as highlighted by 2018 Nobel prize in chemistry for Frances H. Arnold, George P. Smith and Sir Gregory P. Winter, the last two ones "for the phage display of peptides and antibodies" by developing small binding molecules. In this respect, cyclic peptides are of interest as they have more conformational restraints than linear molecules.

Quite successful solubility prediction methods have been developed for small molecules that are most common as drugs (see chapters in this volume). These methods, however, do not work for proteins. There are several reasons. Proteins are much larger, they have lots of groups and protein folding affects the solvent accessibility of the groups. Further, the protein structures are flexible and have a large number of slightly different conformations. Therefore, different approaches are needed for protein solubility prediction. Here, methods for protein solubility and aggregation prediction are introduced. Further, methods to investigate the effects on solubility due to amino acid substitutions (variations) are discussed. This topic is important for the design of changes for protein engineering e.g. to increase protein solubility, production etc.

Protein solubility is discussed here from the perspective of predictions for solubility, aggregation and for variation effects. The principles of the methods are discussed along with performances of the tools. It is apparent that the performances of the methods are not very high, which is due to the complexity of the phenomenon and thereby difficulty in finding good features that would reliably distinguish between the solubility states. One limiting factor has been the small number of experimentally verified cases. However, the field is making progress and there are some rather good methods available.

## Solubility prediction

During the last decade, several computational methods have been developed to predict protein solubility, especially in the context of heterologous protein overexpression (Table 1). These methods utilize different approaches, often in the field of machine learning. Solubility is a complex phenomenon and good predictive features are difficult to find, however, there are some trends such as residue-residue interactions [7] and structural flexibility [8].

Based on examples with known solubility, computer tools have been trained (see e.g. [9]). Protein related parameters such as hydropathy scales and amino acid compositions have been used as features. The best methods claim accuracy of over 80% for two-state predictions of soluble/insoluble. Methods in this category include ccSOL omics [10], DeepSol [11], PaRSnIP [12], Protein-Sol [13], SODA [14], SOLart [15], SOLpro [16], SWI [8] and others.

Most methods are trained on one of two major datasets. Protein Structure Initiative was a large project to determine protein structures *en masse*. Their Structural Biology Knowledgebase [17] contains information for crystallization trials and for (heterologous) protein production and has been used for training many of the tools including ccSOL omics, PaRSnIP, different versions of DeepSol and SWI. eSOL solubility database (http://tanpaku.org/tp-esol/index.php?lang=en) for almost all *Esherichia coli* proteins [18] has been used for some other methods, such as Protein-Sol, SOLart along with *Saccharomyces cerevisiae* protein solubility details [19]. The remaining methods are trained with PON-Sol data [4] or not trained at all.

Although many proteins are poorly soluble their solubility is biologically sufficient as many proteins have very low abundance in cells [1]. Data extracted by DeepSol developers from Structural Biology Knowledgebase indicated that 45.2% out of 129.643 tested inherent and heterologously expressed proteins in *E. coli* were soluble [11].

DeepSol is a deep learning method, ccSOL omics is a support vector machine solution, PaRSnIP utilizes gradient boosting, SOLart random forest, SWI and Protein-Sol are based on weighted scores. Details for the algorithm used in SODA have not been released.

As with any prediction task, the choice of the tool(s) is important. The best comparisons are independent benchmark studies [20, 21], however such studies are not available for any of the prediction tasks discussed in here. Instead, there are some comparisons of methods along with predictor description, including comparisons of four [14], seven [12] and eight [11] tools. The first of these studies indicated SODA to be the best, accuracy 59.2, the second PaRSnIP (accuracy 74.11 and Matthews correlation coefficient (MCC) 0.48) and now defunct PROSO II (accuracy 64.35, MCC 0.31) [22], and in the third DeepSol, PaRSnIP and PROSO II were the best ones with accuracies and MCC values of 0.77/0.55, 0.74/0.48 and 0.64/0.34, respectively.

Analysis of predictions for 57 UDP-dependent glycosyltransferases with 11 predictors [23] is interesting, however, may be biased due to containing proteins just from a single family. The best performing methods in their analysis were SoluProt, DeepSol versions 3, 1 and 2, SolPro and PaRSnIP. Unfortunately, the results are provided only in a format of a figure, thus no numbers are available.

## Aggregation prediction

Aggregation is largely mediated by short sequence stretches of consecutive residues, these regions are typically 15 residues or longer [24]. Almost all human proteins can form self-complementary and thus aggregation-prone segments [25]. However, many proteins do not aggregate and during evolution have adapted to prevent amyloid formation [26]. Chaperones that assist proteins to fold are in central role [27]. Aggregated proteins form either amyloid fibrils, amorphous or native-like deposits. CPAD [28], AmyLoad [29] and AmyPro [30] are databases dedicated for information about protein aggregation. CPAD includes data for amyloid peptides, aggregation prone peptides and aggregation rates while AmyLoad contains amylodogenic sequence information. Proteins containing experimentally verified amyloidogenic regions are collected to AmyPro.

Aggregation predictors can be grouped to two major categories: sequence-based and three-dimensional structure-based (Table 2). The structure-based methods utilize calculations of free energy difference between solution and aggregation phases, β-structure formation propensity, residue exposure and so on. Since protein 3D structures are not always available, sequence-based methods are needed, as well. Machine learning methods use features such as amino acid composition and proportions of certain amino acid types for training. Aggregation predictors include for example AGGRESCAN [31], AGGRESCAN3D [32], AMYLPRED [33], ArchCandy [34], FoldAmyloid [35], MetAmyl [36], PASTA 2.0 [37], TANGO [38], and Waltz [39]. AGGRESCAN3D and ArchCandy are based on three dimensional structures, some of the others use some structural features, as well.

AmylPred and MetAmyl are metapredictors, i.e. use predictions from other tools. RFamyloid is machine learning-based method, Waltz has a position specific scoring matrix, the others are based on physicochemical propensities and other features including information about secondary structural elements, amino acid composition, structural features, physicochemical propensities of amino acids, packing density, hydrogen bonds etc. Neural network tool for amyloid aggregation rate prediction is a related application [40]. Some of the methods can be used both for solubility and aggregation prediction, such as SOLart [15]. SODA, a solubility predictor, utilizes aggregation and disorder propensities [14].

Systematic independent method performance assessments are missing. Some recent studies contain comparisons of several methods. PASTA 2.0 was compared to eight methods, showing the best performance (MCC 0.24) along with AMYLPRED 2 (0.22) and MetAmyl (0.19) [37]. However, the performances are not very high, specificity being clearly better than sensitivity for all the tools, the best Matthews correlation coefficient being only 0.24. Developers of ArchCandy saw really big differences in performances for six predictors [41], their own tool (error rate for amyloid prediction 1.4%) along with PASTA (4.2%), TANGO (5.0%) and Waltz (11.4%) being the best. This test contained only soluble segments of 15 residues or longer. They were collected from three-dimensional protein structures determined with NMR, thus the proteins are soluble, however contained highly flexible regions without ordered structures, typically in the termini. Comprehensive performance assessment would require both positive and negative cases [20, 21], thereby we do not know if the best methods are overpredicting soluble regions and underpredicting aggregation-prone segments.

## Prediction of solubility and aggregation affecting variants

The tools described above are for predictions on entire proteins or polypeptides. Much less effort has been put on predicting the effect of variations on solubility or aggregation. We are looking at tools predicting consequences of amino acid substitutions as there is not enough data for other types of variations. Single amino acid alterations can have profound effects of solubility and lead to diseases, including severe complex V deficiency [42] and cataract [43]. Protein solubility and aggregation are mechanisms in diseases, including cancers [44, 45].

Predictors for solubility affecting variants include CamSol [46], OptSolMut [47], PON-Sol [4], SODA [14] and SolubiS [48]. CamSol uses residue-specific solubility profile. The method is not available as a tool, only the algorithm has been described. OptSolMut has been trained with a small dataset that contains also aggregation cases. Weights for scoring function were optimized with linear programming for 137 cases of single and multiple variants. PON-Sol is a random forest-based machine learning method. It was trained and tested on 406 single amino acid substitutions for which solubility effect have been experimentally determined. It predicts variants into three classes: solubility decreasing and increasing variants and those not affecting solubility. This is a more realistic scenario than binary prediction. In the three-state prediction PON-Sol had correct prediction ratio of 0.597 on cross validation and 0.488 for independent test set (note that random prediction has a score of 0.33). Thus, there is still place for substantial improvements. The method development has been hampered by small number of known solubility-affecting variants. PON-Sol is no more available; however, a new extended and improved version will be released soon. SODA has been recommended to predict variants decreasing solubility [14]. It was developed with PON-Sol data.

These methods can be used for numerous purposes including identification of disease related amino acid substitutions, predictions of solubility of heterologous recombinant protein expression and enhanced crystallizability. Of the aggregation prediction tools, PASTA 2.0 can predict also effects of amino acid substitutions. SoluBis has a somewhat different application, optimization of multiple variants to increase protein solubility [48]. It detects aggregation prone segments and then suggests variants to modify them. So, actually it is an aggregation prevention predictor. It combines predictions from interaction analysis tool FoldX [49], aggregation predictor TANGO [38] and structural analysis with YASARA [50].

## Predictions for protein engineering

Protein properties have complex relations. Several approaches have been tried to improve solubility or prevent aggregation. Electrostatic interactions have been a starting point for one approach [51], stability and aggregation for another [52], and surface patches to avoid aggregation for a third one [53]. SolubiS tries to reduce aggregation propensity [48].

Structural changes designed by four tools have been reviewed in relation to structure-based predictions [54]. The discussed tools included Aggrescan3D, CamSol, Spatial Aggregation Propensity (SAP) and SolubiS. SAP was a proposal to apply molecular dynamics simulations, not an implemented tool [55].

Systematic performance assessments have not been made to these methods. Massively parallel reporter assay (MPRA) of two proteins, TEM-1 β-lactamase, a common antibiotic resistance protein in Gram positive bacteria, and *E. coli* levoglucosan kinase, indicated trade-offs between fitness and solubility [56]. Solubility in this paper was defined as properly folded protein. They used two analysis methods, which revealed whether the protein was folded. In yeast surface display screen the investigated protein had to be folded otherwise the fusion protein was degraded. In twin-arginine translocation-selective export the protein was exported to bacterial periplasm only if correctly folded. They generated >93% of all possible single amino acid variants in the two proteins. Solubility increasing variants were rare, only 4 to 5% had this effect. Many solubility increasing variants affected also some other property. Comparisons to fitness increasing variants revealed that these two features co-occurred very rarely and there were trade-offs between them.

## Conclusions

Computational methods available for the prediction of protein solubility and aggregation were discussed along with tools for engineering solubility or aggregation by introducing amino acid substitutions. Many features and different algorithms have been applied to the available solutions. Although systematic performance assessments have not been performed, it is evident that the methods have widely varying performances. Solubility and avoidance of aggregation are crucial properties for any protein to be used for diagnosis or therapy.

## Figures and Tables

**Table 1.** Protein solubility predictors

Method	URL
Trained with data from Structural Biology Knowledgebase	
ccSOL omics	http://s.tartaglialab.com/update_submission/45568/57e42bea38
DeepSol	https://zenodo.org/record/1162886#.Xoxw_EGxVEY
PaRSnIP	https://github.com/RedaRawi/PaRSnIP
SWI	https://tisigner.com/sodope
Trained with data from eSOL	
ProteinSol	https://protein-sol.manchester.ac.uk/
SOLart	http://babylone.ulb.ac.be/SOLART/
SOLpro	http://scratch.proteomics.ics.uci.edu/
Trained with data from PON-Sol	
SODA	http://old.protein.bio.unipd.it/soda/

**Table 2.** Protein aggregation predictors

AGGRESCAN	http://bioinf.uab.es/aggrescan/
AGGRESCAN3D	https://bitbucket.org/lcbio/aggrescan3d/src/master/
AMYLPRED2	http://aias.biol.uoa.gr/AMYLPRED2/
ArchCandy	https://bioinfo.crbm.cnrs.fr/index.php?route=tools&tool=7&lang=uk
FoldAmyloid	http://bioinfo.protres.ru/fold-amyloid/
MetAmyl	http://metamyl.genouest.org/e107_plugins/metamyl_aggregation/db_prediction_meta.php
PASTA 2.0	http://protein.bio.unipd.it/pasta2/help.html
RFAmyloid	http://server.malab.cn/RFAmyloid/
Tango	http://tango.crg.es/
Waltz	https://waltz.switchlab.org/
